# The elevation in circulating anti-angiogenic factors is independent of markers of neutrophil activation in preeclampsia

**DOI:** 10.1007/s10456-012-9261-5

**Published:** 2012-03-08

**Authors:** Wenda Ramma, Irina A. Buhimschi, Guomao Zhao, Antonette T. Dulay, Unzila Ali Nayeri, Catalin S. Buhimschi, Asif Ahmed

**Affiliations:** 1 University/BHF Centre for Cardiovascular Sciences, Queen’s Medical Research Institute, College of Medicine and Veterinary Medicine, University of Edinburgh, 47 Little France Crescent, Edinburgh, EH16 4TJ UK; 2 Department of Obstetrics, Gynecology and Reproductive Science, Yale University, New Haven, CT 06520 USA

**Keywords:** Soluble endoglin, Soluble Flt-1, Placenta growth factor, Preeclampsia, Neutrophil, Inflammation

## Abstract

**Background:**

Severe preeclampsia is associated with increased neutrophil activation and elevated serum soluble endoglin (sEng) and soluble Flt-1 (sFlt-1) in the maternal circulation. To dissect the contribution of systemic inflammation and anti-angiogenic factors in preeclampsia, we investigated the relationships between the circulating markers of neutrophil activation and anti-angiogenic factors in severe preeclampsia or systemic inflammatory state during pregnancy.

**Methods and results:**

Serum sEng, sFlt-1, placenta growth factor, interleukin-6 (IL-6), calprotectin, and plasma α-defensins concentrations were measured by ELISA in 88 women of similar gestational age stratified as: severe preeclampsia (sPE, n = 45), maternal systemic inflammatory response (SIR, n = 16) secondary to chorioamnionitis, pyelonephritis or appendicitis; and normotensive controls (CRL, n = 27). Neutrophil activation occurred in sPE and SIR, as α-defensins and calprotectin concentrations were two-fold higher in both groups compared to CRL (*P* < 0.05 for each). IL-6 concentrations were highest in SIR (*P* < 0.001), but were higher in sPE than in CRL (*P* < 0.01). sFlt-1 (*P* < 0.001) and sEng (*P* < 0.001) were ≈20-fold higher in sPE compared to CRL, but were not elevated in SIR. In women with sPE, anti-angiogenic factors were not correlated with markers of neutrophil activation (α-defensins, calprotectin) or inflammation (IL-6).

**Conclusions:**

Increased systemic inflammation in sPE and SIR does not correlate with increased anti-angiogenic factors, which were specifically elevated in sPE indicating that excessive systemic inflammation is unlikely to be the main contributor to severe preeclampsia.

## Introduction

Preeclampsia is a pregnancy-specific hypertensive disorder that affects 3–5% of all pregnant women [1]. The exact etiology is unknown, but angiogenic imbalance and systemic inflammation have emerged as leading causes for the clinical signs of the disorder [2–4].

The angiogenic imbalance hypothesis proposed that preeclampsia arises due to loss of vascular endothelial growth factor (VEGF) activity as a result of rise in soluble Flt-1 (sFlt-1) [5]. The loss of VEGF activity due to increased soluble Flt-1 (sFlt-1) has since been demonstrated in preeclamptic women [6–11]. In addition, preeclamptic women have less TGF-β activity due to a rise in soluble endoglin (sEng) [12, 13]. Importantly, these changes precede the maternal disorder. Circulating levels of sFlt-1 and sEng are elevated several weeks prior to the onset of the clinical manifestations of preeclampsia [14, 15], while placenta growth factor (PlGF) is reduced in the first trimester of women who subsequently developed the disorder [16–20]. These observations demonstrate an angiogenic imbalance in preeclampsia.

Many studies have shown that increased systemic inflammation occurs during normal pregnancy and that inflammation is further enhanced in preeclampsia. During inflammatory insults, neutrophils are the first leukocytes to be recruited [21]. Neutrophil counts are increased in normal pregnancy, and further elevated in preeclampsia [22]. Mild neutrophil activation in the maternal circulation occurs in response to circulating syncytiotrophoblastic apoptotic debris that originate from the placenta [23] and was reported to be confined to the maternal circulation in pregnancy-induced hypertension [24]. Activated neutrophils degranulate, releasing numerous products in the circulation. Alpha-defensins are specific to neutrophils, while calprotectin is released by both neutrophils and macrophages. Both markers of neutrophil degranulation are elevated in preeclampsia [22, 25, 26]. Interleukin-6 (IL-6), a pro-inflammatory cytokine secreted by both activated leukocytes and endothelial cells, is also elevated in serum and plasma of women with severe preeclampsia [22, 27, 28].

Both the relationship between anti-angiogenic factors and neutrophil activation and the magnitude of their increase remains unknown. To dissect the contribution of neutrophil activation and anti-angiogenic factors in severe preeclampsia we investigated the relationships between the circulating markers of neutrophil activation/inflammation status (α-defensins, calprotectin and IL-6) and anti-angiogenic factors (sEng and sFlt-1) in severe preeclampsia or systemic inflammatory state during pregnancy.

## Methods and materials

### Patients and biological specimens

We analysed blood samples from 88 women with singleton pregnancies recruited in the Low- and High-Risk Clinics and Labour and Delivery Unit at Yale-New Haven Hospital between December 2004 and March 2010**.** All women were followed prospectively from enrollment until delivery. Women with severe preeclampsia (**sPE**) (n = 45, gestational age (GA) median [interquartile range]: 30 [27–32] weeks) were enrolled at the time of clinical diagnosis. Controls (**CRL**) were healthy normotensive women who had an uncomplicated pregnancy and delivered at term (CRL, n = 27, GA: 29 [26–32] weeks). In addition, samples were collected from a group of pregnant women presenting with symptoms of systemic inflammatory response (**SIR**) syndrome (SIR, GA: 30 [27–32] weeks), who were admitted for clinical work-up targeted to identify the underlying cause. The Yale University Human Investigation Committee approved the research protocol, and written informed consent was obtained from all participants.

Gestational age was established based on last menstrual period and/or early ultrasound evaluation (<20 weeks of gestation) in all cases [29]. Severe preeclampsia was defined based on the American College of Obstetricians and Gynecologists criteria: gestational age >20 weeks, blood pressure of 160 mm Hg systolic or higher or 110 mm Hg diastolic or higher on 2 occasions at least 6 h apart, and/or proteinuria of at least 5 g in a 24-h urine specimen or 3+ or greater on 2 random urine samples collected at least 4 h apart [30]. Other elements of the diagnosis included: IUGR (<10th percentile), persistent neurologic symptoms (headache, visual changes), epigastric or right upper-quadrant pain, pulmonary edema or cyanosis, oliguria (urinary output <500 mL/24 h), serum creatinine >1.2 mg/dL, elevated liver enzymes (greater than two times the normal) and thrombocytopenia (<100,000 cells/μL) [30].

All patients with SIR presented with fever of >38°C (100.4 F) and at least one of the following conditions: maternal leukocytosis (>15,000 cells/mm^3^), maternal tachycardia (>100 beats/min) or fetal tachycardia (>160 beats/min). All SIR patients had a transabdominal amniocentesis to confirm or exclude intra-amniotic infection. In the context of a negative amniotic fluid analysis, a thorough imaging and microbiological workup was conducted which included ultrasonography, MRI, blood, urine and sputum cultures as deemed necessary in the context of additional clinical manifestations. Final diagnoses were as follows: intra-amniotic infection (n = 7), pyelonephritis (n = 3), bloodstream infection (n = 3), gastroenteritis (n = 1), pneumonia (n = 1), appendicitis (n = 1). Serum and plasma were collected during clinical assessment or admission, as previously described [31]. Blood (serum and citrated plasma) were collected by venipuncture, prior to intravenous fluid administration. Serum tubes were allowed to clot at room temperature for 30 min. All samples were spun at 800 g at 4°C for 15 min, the supernatant aliquoted and immediately stored at −80°C until analyzed.

### Immunoassays and other biochemical measurements

Serum sEng, sFlt-1 and PlGF were measured using ELISA according to the manufacturer’s instructions (R&D Systems, Minneapolis, MN). ELISA assays for serum IL-6 (eBioscience, San Diego, CA) and calprotectin (Hycult Biotechnology, Uden, Netherlands), and plasma α-defensins [32] (HNP1-3, Hycult Biotechnology, Uden, Netherlands) were performed according to the manufacturer’s protocol.

### Statistical analysis

Normality testing was performed using the Shapiro–Wilk test. Comparisons between two groups were performed using Mann–Whitney tests or between 3 groups using Kruskal–Wallis on ranks followed by Dunn’s tests as appropriate. For immunoassay results, logarithmic transformations were applied before statistical comparisons were performed. Relationships between variables were explored using Spearman’s Rank order correlations. Comparison between strength of correlations was achieved based on a z statistic. A probability level of <0.05 was considered statistically significant. Exact P values for non-parametric pairwise comparisons were obtained with SPSS (PASW Statistics v.18, IBM Armonk, NY). Multiple stepwise regression analysis was used to explore concurrent relationships between inflammatory and anti-angiogenic markers as dependent variables and demographic or clinical characteristics as independent variables. Variables were entered in the model based on *P* < 0.05 and removed if *P* > 0.1. Statistical analyses were performed with GraphPad Prism (v 4.0; GraphPad Software Inc, La Jolla, CA), SigmaPlot 11.0 (Systat Software Inc, San Jose, CA) or MedCalc (Broekstraat, Belgium) softwares.

## Results

### Demographic, clinical and outcome characteristics

Gestational age at sample collection was not different among the 3 groups (Table 1). Compared to CRL, women with SIR were more likely to be non-Caucasian. Women with sPE had higher blood pressures and proteinuria compared to both non-preeclamptic groups. Twelve of the patients with sPE (29%) had IUGR (<10 percentile). There were significant differences in hematological parameters among groups. Compared to CRL, women with SIR had higher WBC and neutrophil counts and were significantly lymphogenic. Among sPE women, there were wide variations in absolute neutrophil counts (ANC) ranging from 3,000 to 20,000 cells/mm^3^. There was a direct correlation between ANC and 24 h-proteinuria (R = 0.508, *P* = 0.003). There was no relationship between ANC and blood pressure levels or presence of IUGR or HELLP syndrome. As expected, sPE women delivered at earlier gestational ages compared to CRL and SIR.

**Table 1:** Demographic, clinical and outcome characteristics of the study groups

Variable	sPE n = 45	CRL n = 27	SIR n = 16	*P* value
Demographic and clinical characteristics				
Age (years)^†^	27 [20–36]	29 [23–32]	31 [21–38]	0.745
Non-Caucasian race, n [%]^§^	28 [62]	10 [37]	13 [81]	0.013
Weight (kg)^†^	82 [69–100]	74 [63–84]	83 [64–97]	0.377
Nulliparity, n [%]^§^	28 [62]	11 [41]	10 [63]	0.172
Gestational age (weeks)^†^	30 [27–32]	29 [26–32]	30 [28–33]	0.443
Systolic blood pressure (mmHg)^†^	170 [160–180]^a,b^	114 [101–127]	108 [101–124]	<0.001
Diastolic blood pressure (mmHg)^†^	101 [92–110] ^a,b^	68 [60–76]	60 [51–72]	<0.001
Proteinuria—urinary dipstick^†^	3 [2–3] ^a,b^	0 [0–0]	0 [0–0]	<0.001
24 h-protein excretion (g/24 h)^†^	2.6 [1.3–5.3]	NA	NA	NA
Neurological manifestations, n [%]^§^	22 [49]^a,b^	0 [0]	0 [0]	<0.001
IUGR, n [%]^§^	12 [27]^a,b^	0 [0]	0 [0]	0.001
HELLP syndrome, n [%]^§^	13 [29]^a,b^	0 [0]	0 [0]	<0.001
Laboratory characteristics				
WBC (×1,000/mm^3^)^†^	11.7 [8.6–14.7]	10.5 [8.3–12.5]^b^	13.6 [11.8–20.8]^a^	0.006
Hemoglobin (g/dL)^†^	12.3 [11.2–13.2]^b^	12.2 [11.5–13.0]^b^	10.8 [10.2–12.2]^a^	0.013
Hematocrit (%)^†^	36.1 [33.4–38.8]^b^	33.9 [33.6–37.2]^b^	32.7 [29.6–35.3]^a^	0.004
Differential count				
Neutrophils (%)^†^	75 [65–84]^b^	76 [67–79]^b^	82 [80–87]^a^	0.009
Lymphocytes (%)^†^	17 [11–27]^b^	18 [15–23]^b^	9 [7.3–12]^a^	<0.001
Monocytes (%)^†^	6 [4–8]	5 [4–7]	6 [4–8]	0.840
ANC (×1,000/mm^3^)^†^	8.9 [6.2–11.9]^b^	7.6 [5.8–10.1]^b^	11.6 [10.0–16.2]^a^	0.002
Outcome characteristics				
Delivery <34 weeks, n [%]^§^	42 [93]^a,b^	0 [0]^b^	7 [44]^a^	<0.001
Gestational age at delivery (weeks)^†^	29 [28–32]^a,b^	39 [38–40]^b^	36 [29–39]^a^	<0.001

Gestational age equates to the gestational time at which blood was taken and the time of delivery is given as the “gestational age at delivery”

*IUGR* intrauterine growth restriction; *HELLP* Hemolysis, ELevated liver enzymes and Low Platelets; *WBC* white blood cell count; *ANC* absolute neutrophil count

^†^ Data presented as median [interquartile range] and analyzed by Kruskal–Wallis ANOVA

^§^ Data presented as n [%] and analyzed by Chi square test

^a^ *P* < 0.05 versus CRL; ^b^ *P* < 0.05 versus SIR

### Neutrophil activation and inflammation

**Fig. 1:**
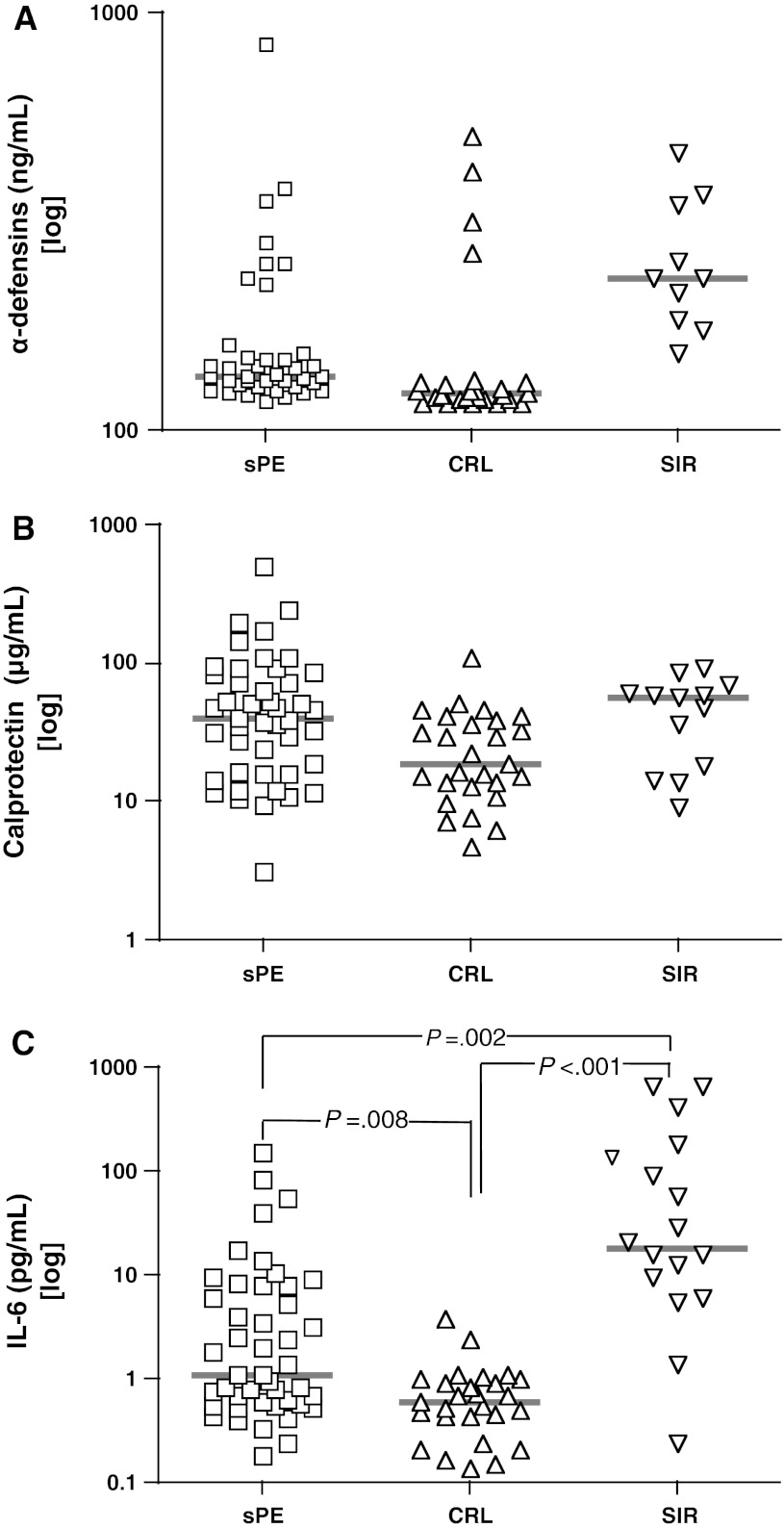
Scatterplots of the markers of neutrophil activation and the inflammatory cytokine interleukin-6. Levels of α-defensins (**a**), calprotectin (**b**) and interlukin-6 (IL-6) (**c**) in women with severe preeclampsia (sPE, n = 45), pregnant controls (CRL, n = 27) and women with systemic inflammatory response syndrome (SIR, n = 16). The *thick red line* represents the groups’s median. Statistical analysis: Kruskal–Wallis ANOVA followed by multiple post hoc Dunn’s tests

Neutrophil activation, assessed by α-defensins and calprotectin concentrations in the maternal circulation, showed that the median plasma level of α-defensins in sPE was only increased by 1.5-fold compared to CRL (Table 2, Fig. 1). Women with SIR exhibited a further significant increase in plasma α-defensins compared to both sPE and CRL groups (Fig. 1). The median calprotectin concentration was increased by approximately twofold in sPE compared to CRL, reaching a similar level to that observed in SIR (Fig. 1). Inflammation in sPE group was significantly higher than CRL, as demonstrated by the twofold increase serum IL-6 levels in sPE compared to the CTL group. The SIR group exhibited significantly higher IL-6 compared to both sPE and CRL groups (Fig. 1 and Table 2).

**Table 2:** Levels of inflammatory and angiogenic markers in maternal circulation

Variable	sPE n = 45	CRL n = 27	SIR n = 16	*P* value
Inflammatory markers				
α-Defensins (pg/mL)^†^	135.7 [127.8–153.7]^a,b^	122.9 [119.3–130.2]^b^	233.0 [182.3–351.3]^a^	<0.001
Calprotectin (μg/mL)	40.7 [16.2–86.4]^a^	18.8 [13.0–39.4]	56.8 [16.5–65.3]^a^	0.010
Interleukin-6 (pg/mL)	1.1 [0.6–7.9]^a,b^	0.6 [0.4–1.0]^b^	18.6 [7.1–162.1]^a^	<0.001
Pro and anti-angiogenic markers				
sEndoglin (ng/mL)	70.1 [41.3–109.4]^a,b^	4.3 [3.5–6.1]	4.9 [7.3–7.0]	<0.001
sFlt-1 (ng/mL)	21.5 [15.2–30.5]^a,b^	1.5 [1.1–1.8]	2.5 [1.5–4.1]	<0.001
PlGF (pg/mL)	65.5 [33.0–101.2]^a,b^	432.9 [359.6–815.2]	203.0 [142.0–536.8]	<0.001
sFlt-1/PlGF ratio	384.6 [180.3–641.9]^a,b^	3.4 [1.9–6.1]	9.2 [4.4–13.6]	<0.001

^†^ Data presented as median [interquartile range] and analyzed by Kruskal–Wallis ANOVA followed by multiple post hoc Dunn’s tests

^a^ *P* < 0.05 versus CRL

^b^ *P* < 0.05 versus SIR

### Angiogenic factors

**Fig. 2:**
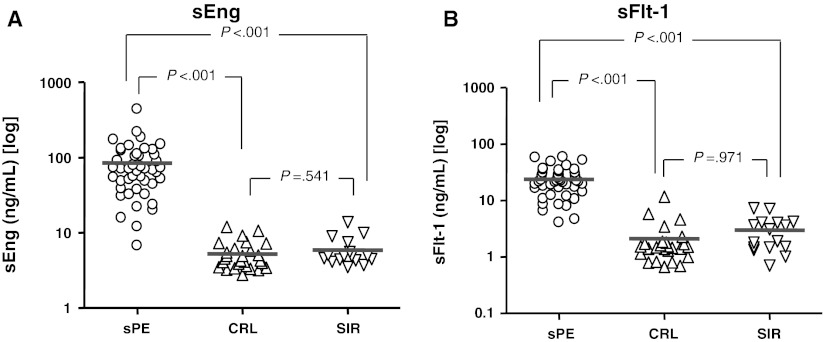
Scatterplots of the anti-angiogenic factors. Levels of serum soluble endoglin (sEng) (**a)**, soluble fms-tyrosine kinase receptor-1 (sFlt-1) **(b)** in women with severe preeclampsia (sPE, n = 45), pregnant controls (CRL, n = 27) and women with systemic inflammatory response syndrome (SIR, n = 16). The *thick red line* represents the groups’s median. Statistical analysis: Kruskal–Wallis ANOVA followed by multiple post hoc Dunn’s tests

Compared to CRL, the sPE group had an ~20-fold increase in the median serum sEng and sFlt-1 concentrations (Fig. 2) accompanied by decreased PlGF (Table 2). Importantly, despite the elevated inflammatory status in SIR (Fig. 1), sFlt-1 and sEng concentrations in the SIR group were similar to CRL (Fig. 2).

### Inter and intra-process relationships

**Fig. 3:**
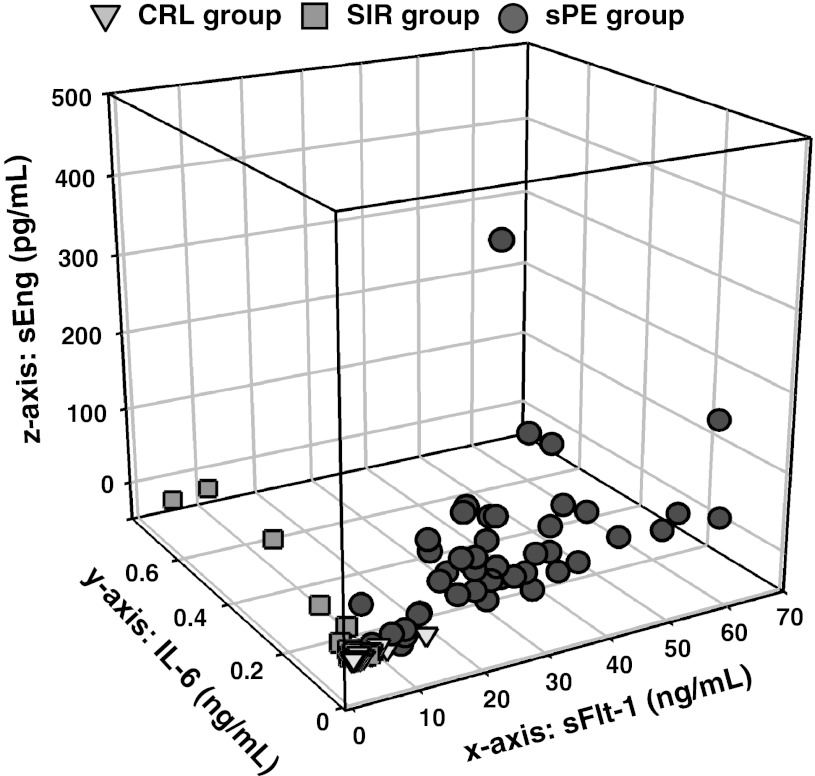
Three-dimensional scatterplot of the study population. Each case is represented by its soluble fms-tyrosine kinase receptor-1 (sFlt-1, *x* axis), interlukin-6 (IL-6, *y* axis) and serum soluble endoglin (sEng, *z* axis). *Red circles* cases with severe preeclampsia (sPE, n = 45); *green squares* cases with systemic inflammation (SIR, n = 16); *yellow triangles* pregnant controls (CRL, n = 27)

To further dissect the relationships between the two biological processes (inflammation and anti-angiogenic status) in preeclamptic women, we performed correlation analyses of biomarkers representative for each process (intra-process correlations) using the whole cohort (3 groups). We then analysed intra and ieter-process relationships within the sPE group. Whole cohort correlations showed that inflammatory markers α-defensins, calprotectin and IL-6 correlated strongly with each other. This indicated that they were indeed representing measurements of the same biological process (Table 3A). Similarly, the placental angiogenic factors (sEng, sFlt-1 and PlGF) also correlated with each other, suggesting that their release in the circulation likely occurs via related pathways (Table 3B). Significant positive intra-process correlations between the angiogenic markers, as well as the markers of inflammation, were also observed within the sPE group (Table 4). However, inter-process correlations in the sPE group revealed lack of correlation between the markers of inflammation and an anti-angiogenic state. The only inter-process correlation that reached statistical significance was between calprotectin and PlGF (inverse association, *P* = 0.011). A graphical representation of these observations is shown in Fig. 3. Each case in our cohort is plotted in the 3D space relative to its *x*, *y* and *z* coordinates represented by its sFlt-1, sEng and IL-6 concentration, respectively. As shown, the only variation along the *y* axis (depth: IL-6) is observed among patients in the SIR group (green squares). In contrast, sPE cases (red circles) scatter in the front plane of the graph delineated by their variation in the *x*- (length: sFlt-1) and *z*- (height: sEng) coordinates. As expected, CRL cases (yellow triangles) cluster together around the point of origin indicating minimal variation in the 3 analytes.

**Table 3:** Intra-process relationships among inflammatory (A) versus angiogenic markers (B)

	α-Defensins	Calprotectin	IL-6
A. Inflammatory markers			
α-Defensins	**r** **=** **1.000**		
Calprotectin	*r* = *0.563*	**r** **=** **1.000**	
	*P* < *0.001*		
IL-6	*r* = *0.544*	*r* = *0.453*	**r** **=** **1.000**
	*P* < *0.001*	*P* < *0.001*	

	sFlt-1	sEng	PlGF
B. Angiogenic markers			
sFlt-1	**r** **=** **1.000**		
sEng	*r* = *0.852*	**r** **=** **1.000**	
	*P* < *0.001*		
PlGF	*r* = −*0.692*	*r* = −*0.778*	**r** **=** **1.000**
	*P* < *0.001*	*P* < *0.001*	

Data presented as Spearman correlation coefficients and *P* values in the entire cohort (n = 88). Italicized values depict non-significant correlations (*P* < 0.05). Perfect correlations are noted by the bold values

**Table 4:** Comparison of inter- vs intra-process relationships in sPE group (n = 45)

Markers	α-Defensins	Calprotectin	IL-6	sFlt-1	sEng	PlGF
α-Defensins	**r** **=** **1.000**					
Calprotectin	*r* = *0.603*	**r** **=** **1.000**				
	*P* < *0.001*					
IL-6	*r* = *0.645*	*r* = *0.372*	**r** **=** **1.000**			
	*P* < *0.001*	*P* = *0.012*				
sFlt-1	r = −0.301	r = −0.209	r = −0.122	**r** **=** **1.000**		
	*P* = 0.062	*P* = 0.167	*P* = 0.423			
sEng	r = 0.145	r = 0.227	r = 0.080	*r* = *0.391*	**r** **=** **1.000**	
	*P* = 0.376	*P* = 0.133	*P* = 0.597	*P* = *0.008*		
PlGF	r = −0.211	*r* = −*0.379*	r = −0.226	r = −0.023	*r* = −*0.552*	**r** **=** **1.000**
	*P* = 0.196	*P* = *0.011*	*P* = 0.135	*P* = 0.882	*P* < *0.001*	

Data presented as Spearman correlation coefficients and *P* values. Italicized values depict non-significant correlations (*P* < 0.05). Other values indicate non-significant correlations (*P* > 0.05). Perfect correlations are noted by the bold values

## Discussion

The study examined the contribution of systemic inflammation and anti-angiogenic factors in preeclampsia by investigating the relationships between circulating markers of neutrophil activation/inflammation and anti-angiogenic factors in severe preeclampsia and systemic inflammatory status during pregnancy. The results reveal two important findings. First, this case–control study demonstrates that increased neutrophil activation and the release of anti-angiogenic factors in preeclampsia occur at different magnitudes in severe preeclampsia. Second, higher levels of anti-angiogenic sFlt-1 and sEng are specific to severe preeclampsia, and did not occur in women with systemic inflammatory state during pregnancy.

The positive correlation between α-defensins and calprotectin demonstrates that neutrophil degranulation occurs in severe preeclampsia. These markers of neutrophil activation were positively correlated with IL-6, indicating that neutrophil activation is accompanied by inflammation at the time of the clinical manifestation of the disease. The cohort of severe preeclampsia patients exhibited significantly elevated circulating sEng and sFlt-1. However, there was no meaningful relationship between the increase in sEng or sFlt-1 and neutrophil activation, as determined by α-defensins or calprotectin and IL-6 release, in severe preeclampsia. More importantly, patients with clinically relevant systemic inflammatory state during pregnancy did not exhibit elevated maternal sEng and sFlt-1 despite raised levels of α-defensins, calprotectin and IL-6. This indicates that the rise in these anti-angiogenic factors is specific to preeclampsia and their release in the maternal circulation is unlikely to be triggered by neutrophil activation.

Activation of neutrophils and their degranulation result in the generation of reactive oxygen species and oxidative stress. Many studies have proposed that oxidative stress could be the main placental problem leading to preeclampsia [33]. Recently, Redman and Sargent suggested that oxidative stress could induce sFlt-1 and sEng release via nuclear factor kappa-B (NFκB) to a similar or greater extent as hypoxia [4]. In our study, women with preeclampsia had a 20-fold increase in serum sEng and sFlt-1 and approximately a twofold increase in markers of neutrophil activation (α-defensins and calprotectin) and the pro-inflammatory cytokine, IL-6. This is consistent with the results of similar studies on sEng [15], sFlt-1 [14], α-defensins [34], calprotectin [25, 35] and IL-6 [36]. Our findings extend the results of previous studies by demonstrating that neutrophil activation is not associated with increases in sEng or sFlt-1 in severe preeclampsia. To strengthen these findings, we included a group of pregnant patients with systemic inflammation in the absence of preeclampsia. This group failed to show any elevations in circulating sEng or sFlt-1, despite heightened systemic inflammation, indicating that the rise of anti-angiogenic factors is specific to preeclampsia and independent of systemic inflammation. Although, the present results conflict with those of an earlier study, which had shown a positive correlation between calprotectin and sEng in preeclampsia [37], the previous results are likely to be the consequence of gestational age differences among groups, as acknowledged by the authors themselves [37]. To eliminate this possible confounder, our patient groups were matched for gestational age at sample collection and the correlation between the two processes was limited to the severe preeclampsia group.

The present study suggests that neutrophil activation is unlikely to be directly involved in the release of maternal anti-angiogenic factors in severe preeclampsia. This conclusion is supported by recent studies demonstrating that complement activation was not associated with the release of angiogenesis-related factors in preeclamptic women [38, 39]. Interestingly, Girardi and co-workers showed that complement activation induces the release of sFlt-1 from monocytes and causes abnormal placental development and fetal death in mice [40]. Thus, it is possible that inflammatory mediators may have local autocrine or paracrine effects, which could amplify signaling pathways and enhance the autocoid production of anti-angiogenic factors or their actions. However, compliment activation did not cause the classical symptoms of preeclampsia (increased blood pressure and proteinuria) in the murine model studied by Girardi and co-workers. This suggests that a threshold concentration of sFlt-1 in the maternal circulation is critical to induce preeclampsia-like symptoms, and that the amount produced by complement-mediated activated monocytes was insufficient to elicit the classical signs of preeclampsia. Indeed, the dose-dependent effect of sFlt-1 is illustrated by the fact that neutralization of sFlt-1 below a critical threshold eliminates the signs of preeclampsia in mice [10].

A recent study showed that serial extraction of sFlt-1 from the plasma of severe preeclamptic patients by apheresis reduced circulating sFlt-1 and stabilized maternal blood pressure, prolonging gestation in preeclamptic women [41]. This observation provides strong evidence of the role of anti-angiogenic factors as the likely candidates for severe preeclampsia. In contrast, the hypothesis that systemic inflammatory state per se causes preeclampsia is somewhat at odds with clinical experience of corticosteroids used to accelerate fetal lung maturation. A prospective double-blind randomized clinical trial of betamethasone confirmed the known beneficial effects of this treatment on neonatal outcome, but failed to show any beneficial effect to the preeclamptic mother [42].

In conclusion, our study shows that there was no meaningful relationship between neutrophil activation and angiogenic imbalance in severe preeclampsia. Importantly, the absence of increase anti-angiogenic factors in non-preeclamptic pregnant patients with elevated systemic inflammatory state indicates that it is unlikely that neutrophil activation play a central role in inducing the release of sEng or sFlt-1 during pregnancy. More importantly, since the increase in anti-angiogenic factors, above a critical threshold, is specific to preeclampsia, preeclampsia can be defined as a disease where anti-angiogenic factors disrupt the angiogenic balance, required for healthy pregnancy. In contrast, inflammation is consistently present in a number of pregnancy complications and it is very unlikely that it is the main or even the sole cause of preeclampsia. Our study focused on severe preeclampsia, as these patients have the greatest disease burden for maternal and fetal morbidity and mortality. Additional research is needed to determine whether these results also apply to mild preeclampsia. Larger longitudinal population-based studies of pregnant women will be needed to definitively ascertain whether angiogenic imbalance or excessive inflammation is the cause preeclampsia, and whether these relationships differ according to disease severity.
